# Body mass index and dental caries in children and adolescents: a systematic review of literature published 2004 to 2011

**DOI:** 10.1186/2046-4053-1-57

**Published:** 2012-11-21

**Authors:** Merrilyn Hooley, Helen Skouteris, Cecile Boganin, Julie Satur, Nicky Kilpatrick

**Affiliations:** 1 School of Psychology, Deakin University, 221 Burwood Highway, Burwood, Melbourne, Victoria, 3125, Australia; 2 Melbourne Dental School,Faculty of Medicine Dentistry and Health Sciences, The University of Melbourne, 720 Swanston Street, Melbourne, 3010, Australia; 3 Department of Paediatrics, Faculty of Medicine, Dentistry and Health Sciences, The University of Melbourne, 720 Swanston Street, Melbourne, 3010, Australia; 4 Murdoch Children’s Research Institute, Royal Children's Hospital, Flemington Road, Parkville, Victoria, 3052, Australia

**Keywords:** Early childhood caries, Childhood obesity, Review, Dental caries, Obesity, Overweight

## Abstract

**The objective:**

The authors undertook an updated systematic review of the relationship between body mass index and dental caries in children and adolescents.

**Method:**

The authors searched Medline, ISI, Cochrane, Scopus, Global Health and CINAHL databases and conducted lateral searches from reference lists for papers published from 2004 to 2011, inclusive. All empirical papers that tested associations between body mass index and dental caries in child and adolescent populations (aged 0 to 18 years) were included.

**Results:**

Dental caries is associated with both high and low body mass index.

**Conclusion:**

A non-linear association between body mass index and dental caries may account for inconsistent findings in previous research. We recommend future research investigate the nature of the association between body mass index and dental caries in samples that include a full range of body mass index scores, and explore how factors such as socioeconomic status mediate the association between body mass index and dental caries.

## 

Obesity and dental caries are both multifactorial diseases that impact children’s health and psychosocial development [1,2]. Both conditions contribute substantially to health expenditure; for example, the estimated total annual cost of obesity to Australian society in 2008 was $58.2 billion [3], and recent estimates suggest that being overweight or obese is the third highest contributor to disability-adjusted life years (DALYs; 7.5%) [4]. Dental disease ranks as the second most expensive disease in Australia (second to cardiovascular disease) and absorbs 6.2% of the total recurrent expenditure in health, behind hospital services (39.3%), medical services (18.7%), and medications (14.0%) [4]. Obesity and dental caries share common, modifiable, influences such as diet and lifestyle. Recent national data from Sweden [5] suggest a positive correlation between dental caries and Body Mass Index (BMI), and showed that obesogenic behaviour such as snacking in early childhood predicted caries development in adolescence. BMI is widely used as a surrogate measure for obesity because it corrects for an individual’s height in relation to weight, and is a commonly used indicator of can indicate nutritional status. Given that dental caries rates and BMI both measure diet-related health outcomes, an association between the two is not surprising. Changes to diet and lifestyle since the mid 1990s, such as increased affluence and access to high caloric carbohydrate-rich foods and drinks, may help to account for the rising prevalence in dental caries and obesity since that time [6,7]. However, not all studies have found a positive association between BMI and dental caries; some studies suggest that there is no relationship (for example, [8]) and others show an inverse relationship (for example, [9]).

To our knowledge there has been only one systematic review examining the relationship between obesity and dental caries [10]. This review included only seven studies published between 1984 and 2004, only five of which included a pediatric sample. Of the three A grade studies, one found a positive correlation between dental caries and BMI in a sample of 842 children aged 6 to 11 years [11]. Another found no correlation in a sample of over five thousand 3-year olds [12], while the third was not able to predict future dental caries experience on the basis of BMI status in more than five hundred children (aged 5 to 13 years) [13].

Since publication of this review [10] there has been increased interest in the association between dental caries and BMI. The objective of the current paper was to provide an updated review of the evidence in this emerging area of research. Our systematic review addressed the following specific questions:

1.What do studies reveal about the association between dental caries and BMI in children and adolescents?

2.What are the methodological limitations of the current approaches to investigating the development of both dental caries and obesity and what may be valuable directions for future research?

### Methods

#### Search strategy

The authors searched Medline, Web of Science (ISI), Cochrane, Scopus, Global Health and the Cumulative Index to Nursing and Allied Health (CINAHL) libraries. Additionally, a lateral approach involving a review of reference lists in papers identified was undertaken. Search terms were Obesity OR overweight OR BMI or body mass index OR weight AND Caries or dental health OR oral health OR dmf* OR teeth decay* OR cavities OR cavities. AND Child* OR adol* OR preschool OR toddlers OR pediat* OR paediat*

We did not initially include search terms covering “malnutrition”, but we found no additional studies meeting inclusion criteria after conducting a forensic search using the terms ‘malnutrition OR malnourish*” in addition to our original dental search terms. We included relevant non-English language studies and translated them in to English. To explore development since Kantovitz *et al*. article [10] the search was limited to papers published from January 2004 until June 2011. Studies were included if they satisfied the following three criteria:

1. Measured caries rates, most commonly by variations of the number of decayed (Dd), missing (Mm), filled (Ff) surfaces (Ss) or teeth (Tt) index or presence/absence of caries (that is, DMFT/dmft >0). However studies that categorized teeth conditions (for example, levels of caries experience: frank caries, filled teeth, white spots and no caries [14]) or described dental health (for example, cavity now or ever, filling, tooth pulled, and overall dental health [15]) were also included in the review.

2. Measured some form of weight-to-height ratio to estimate body fat. This was most commonly estimated using BMI, but body fat index (DXA [16]) and Division of Nutrition, Thai Ministry of Public Health standards using weight for height in Thai children [17] were also used.

3. Assessed the relationship between dental caries and BMI in children and adolescents to age 18 years.

#### Methodological quality

Studies were evaluated on the basis of a number of criteria to assess quality of methodology. These criteria are summarized in Table 1 and Additional file 1. The first criterion was representativeness of sample, and studies were ranked 1 (highest) to 4 (lowest) as follows: 1) sample involved forms of stratification or cluster sampling of countries or districts that ensured a representative range of socioeconomic strata; 2) sample represented cities or towns using some form of cluster sampling (for example, of schools); 3) sample of convenience with some randomization involved in selection of participants; and 4) sample of convenience without random selection (for example, patients of obesity clinics, dental hospitals). The second criterion was whether or not an attempt had been made to control potential confounding variables; studies were scored on a yes/no basis (Yes = 1; No = 2). The third criterion assessed the quality of assessment of the child weight-to-height (for example, BMI) and dental caries. Measures of child weight-to-height were scored for objective measurement using standardized equipment (score 1) or other (score 2). Scores for the quality and sensitivity of the dental examination ranged from 1 (highest) to 5 (lowest; see Table 1). Rating 1 examinations were conducted within a dental surgery with mirror and probe by a qualified dentist, dental student, or hygienist and included radiographs. Rating 2 involved dental examinations within a dental surgery with mirror and probe by a qualified dentist, dental student, or therapist/hygienist but did not include radiographs. Examinations Rated 1 or 2 were performed under optimal lighting conditions with dry field available, which improves detection rates and enabled the detection of initial caries lesions. Rating 3 involved examinations that were performed under field conditions, which typically rated caries at the cavity level. Most examinations at this level were carried out with natural lighting, blunt probe and mirror (that is, following WHO criteria), and may have been undertaken by someone other than a qualified dental professional. Rating 4 involved visual examination involving mirror only, and Rating 5 involved parent or self report. Under field conditions obtaining a dry field is difficult although some studies used cotton rolls/pellets; a lowercase superscripted ‘a’ with the rating scores of 3 or 4 denotes these studies. Calibration exercises were undertaken in all studies involving dental examinations.

**Table 1:** Criteria used for rating studies

	**Sample**	**Attempt made to control confounds?**	**BMI measure**	**Dental caries measure**
1	Stratification/cluster sampling use to obtain sample representative of countries/districts	Yes	Standardized	Dental surgery
				Mirror and probe
				Optimal lighting and dry field
				Radiographs
				Dentally qualified examiner calibration
2	Some form of cluster sampling use to obtain sample approximately representative of towns	No	Non-standardized	Dental surgery
				Mirror and probe
				Optimal lighting and dry field
				Dentally qualified examiner calibration
3	Convenience sample with some randomization			Field clinic
				Mirror and probe calibration
4	Convenience sample without randomization			Field clinic
				Visual inspection calibration
5				Parent report

BMI, body mass index.

The potential range of scores following assessment of methodological quality ranged from 4 to 13. Studies scoring 4 to 5 were rated A, 6 to 7 rated B, 8 to 10 rated C and 11 to 13 rated D.

### Results

The flow diagram of the processing of search results is shown in Figure 1. Forty-seven papers met the selection criteria and were included in the review. However, two papers [5,18] reported the same data and were combined for the purpose of this review, while two other papers [19,20] reported findings of two septe studies. A total of 48 studies were therefore included, (excluded papers and reasons for exclusion are presented in Additional file 2). Only two studies were also cited in Kantovitz *et al*. review [8,11].

**Figure 1:**
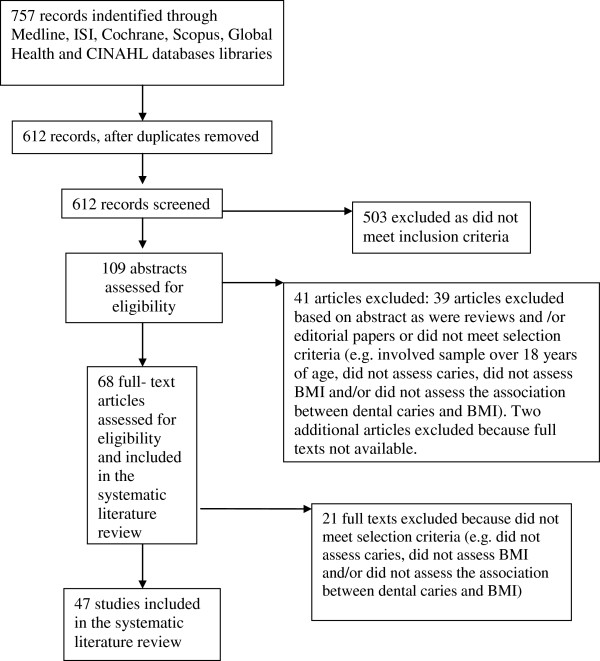
The flow diagram of processing of search result.

Three main patterns of relationships were found between dental caries and BMI: 23 of the 48 studies found no association between BMI and dental caries, 17 found a positive relationship between BMI and dental caries, and nine found an inverse relationship. One additional study found dental caries was associated with both high- and low- BMI [21]; that is, a U-shaped pattern, and another found inconsistent patterns across age cohorts; specifically, an association between lower dental caries and high BMI in two age cohorts, and no association in four cohorts [19]. In order to explore possible explanations for these differences in patterns, the results of the 48 studies were septed and evaluated on the basis of the nature of the relationship between caries and BMI. That is, positive, negative and no association are summarized in Tables 2, 3 and 4 respectively. The study finding that dental caries is associated with both high and low BMI [16] is included in both Tables 2 and 3 and the study finding no association and a negative association between dental caries and BMI depending on the age cohorts is included in Tables 3 and 4. Table 5 summarizes the patterns that emerged within each set of studies.

**Table 2:** Positive association between dental caries and BMI

**Authors**	**Country**	**Design**	**N**	**Age**	**Sampling**	**Dental rating**	**HDI**	**Dental measure**	**Significant cariogenic risk factors**	**Sample demographics**
Alm (2008) and Alm et al. (2008)	Sweden	Prospective longitudinal	402 at age 15 years	1-15; data analysed at age 15 years	Four of the 13 districts of child welfare centres in the Municipality of Jonkoping	1	10	D_ia_ (Initial caries) D_ma_ (Manifest caries) D_i+m_FA or DF_a_ (Total approximal caries prevalence fillings)	Higher approximal dental caries in overweight and obese adolescents than normal and underweight. Caries experience at 15 years predicted by early childhood caries experience at age 3, plaque on incisors at age 1 year, infrequency of tooth brushing with fluoride toothpaste , consumption of caries-risk snacks more than three times a day at age 1 years, consumption of sweets < once per week at age 3 years, parents were born abroad, parents’ poor attitudes to dental health and psychosocial factors	**Initial caries**: 86% of total caries experience **Approximal** caries prevalent in 67% of 15 yr old children **Mn Di+mFa at 15 yrs** Overall sample=3.21 (SD=3.95) Underweight = 2.94 (SD=3.62) Normal weight = 4.64 (SD=5.15) Overweight = 4.18 (SD=5.14) Obese = 6.29 (SD=5.04) **Mn DmFa at 15 yrs** Overall sample 0.42 ( SD=1.13) Underweight = 0.35 ( SD=−0.89) Normal weight = 0.83 ( SD=1.93) Overweight = 0.84 ( SD=2.02) Obese = 0.79 ( SD=1.63) **BMI:** Overweight: 16% Obese: 4% Low-normal weight: 84%
Alm et al. (2011)	Sweden	L &CS	Time 1 (aged 3): 525 Time 2 (aged 6): 506 Time 3 (aged 15): 402	1-15	Four of the 13 districts of child welfare centres in the Municipality of Jonkoping	1	10	Caries (Initial and manifest) prevalence: def in children 3 and 6 years D_i+m_FSa Manifest caries prevalence: (D_m_a)	At 3 years: No association At 6 years: Higher prevalence of manifest caries in overweight/obese At 15 years: Higher rates of proximal caries/fillings in overweight/obese	**BMI:** ** 3 years:** Normal weight: 86% Overweight: 12% Obese: 3% ** 6 years:** Normal weight: 82% Overweight: 14% Obese: 4% ** 15 years:** Normal weight: 84% Overweight: 12% Obese: 4%
Bailleul-Forestier et al. (2007)	France	CC	82	12-18	Case-match control: Treatment program for severe obesity matched for age, gender and parental socio-occupation	3 Cavity level	20	DMFT – ordinal ranking 1-8	Significant association between high dental caries and increased obesity.	**Caries prevalence**: Obese – 90% Control - 80% **Mn DMFT** Obese 6.9 (SD=4.1) Control: 4.3 (SD=3.5) **Mn BMI:** Obese group: 40.6 (SD= 7.3) Control: 19.8 (SD=2.1)
Costacurta et al. (2011)	Italy	CS	107	6-12	Paediatric Dentistry Unit of PTV Hospital, University of Rome “Tor Vergata”	1	24	Dmft/DMFT	Child physical status measured as (i) Fat Mass% (FM; using Dual energy X-ray), and (ii) BMI Children with higher body fat mass (FM%) had higher DMFT/dmft rates than those with normal FM, but compble dmft/DMFT rates with underweight children using FM%-DXA No association found using BMI (McCarthy or WHO cut-offs) as obesity estimate	**Caries Prevalence:** 83.18% **Total Mn dmft**= 2.07 (SD=1.21) by FM% (WHO cut offs) Underweight = 2 (SD=0.61) Normal weight = 1.2 (SD=0.36) Overweight = 1.95 (SD=0.25) Obese = 2.40 (SD=0.52) **Total Mn DMFT**=2.55 (*SD=*2.02) by FM% (WHO cut offs) Underweight = 3.6 (SD=2.7) Normal weight = 1.88 (SD=1.48) Overweight = 1.74 (SD=1.68) Obese = 3.10 (SD=2.11) **Physical status (FM% -Who cut-offs)** Underweight = 4% Healthy weight = 22% Overweight = 21% Obese = 51%
Gerdin et al. (2008)	Sweden	L	2303	4-12	Retrospective archival study of children in single county	1 manifest caries only	10	Deft (6 yrs) DFT (10–12 yrs) DFSa (approximal surfaces of permanent teeth)	Dental caries higher prevalence in obese than non-obese At 12-years of age, children who had a history of obesity at 4-yrs of age had higher rates of caries than children who had history of healthy weight at 4-years of age. Obese/overweight children at 4 yrs old and who remained overweight / obese at 5, 7 and 10 years of age had significantly more approximal carious surfaces (DFSa) than normal weight children in all age groups Overweight / obese children at 4 yrs old but with normal weight at 5, 7 and 10 yrs of age had significantly fewer approximal carious surfaces than children with normal weight from 4 to 10 years of age Gender: Girls at 10 and 12 yrs old had more caries affected teeth than boys SES: Caries prevalence decreased with increasing SES	**Caries Prevalence:** Age 6= 31% Age 10= 23.1% Age 12= 32% **Mn Dft/DFT:** Age 6= 3.8 Age 10= 1.9 Age 12 = 2.1 **BMI:** ** Age 4:** Normal BMI= 87.2% Overweight= 10.8% Obese= 2.0% ** Age 5:** overweight/obese = 14.2% ** Age 6:** Not reported ** Age 7:** overweight/obese = 17.1% ** Age 10:** Overweight: 17.5% Obese= 4.3%
Hilgers et al. (2006)	USA	CS	178 sample of convenience	8-11	Convenience sample – participants of dental treatment program Smile Kentucky – dental needs with no dental insurance	1	4	Interproximal caries in primary and permanent molars Ordinal ranking, 1=incipient, 2=dentine involvement, 3= pulpal involvement; 4= nonrestorable/missing **Severity index** calculated by averaging primary and permanent tooth scores (**c-avg and C-avg**)	Higher permanent molar caries average associated with higher BMI	**C-avg** (severity measure for permanent teeth) ranged from 0 to 4.0 Low BMI:C-avg = 0.08 (0.06) Normal BMI: C-avg 0.19 (0.05) High BMI: C-avg= 0.51 (0.09) **BMI** Ranged from 11.43-35.7
Hong et al. (2008)	USA	CS	1507	2-6	NHANES (1999–2002)	2	4	Dft 0, 1–5, >5 teeth	Higher caries rates significantly associated with higher BMI in 5–6 yr olds and in Hispanic and non-hispanic blacks S-ECC: Logistic regression found BMI did not predict of caries experience but Age and poverty index did	**Caries prevalence 42%** Mn dft 1.79(.09) **BMI** Mn BMI 16.2 (.01) Underweight 4.2% Normal 73.9% At risk 11.3% Overweight 10.6%
Ismail et al. (2009)	USA	L & CS	788	0-5	A two-stage area probability selection of representative sample of low-income African-American in Detroit Michigan. Dyads tested in 2002–3 and 2004-5	2	4	Non-cavitated lesions: (d1-2) Cavitated/dentinal lesions: (d3-6) Filled lesions (f); missing lesions (m) d3-6mfs; d1-6mfs	Higher caries (dmft:1–6) associated with higher weight-for-age. For d1-6mfs: higher consumption of soda drinks, older child age, higher weight-for-age, visiting a dentist for treatment, higher baseline caries level of the child and caregiver, fatalistic belief of the caregiver, and living in relatively disadvantaged low-income neighbourhood.	Almost 25% of children had low weight for age
Marshall et al. (2007)	USA	L	427	1-11	IOWA fluoride study	3- cavity level	4	Caries experience dichotomised =/>0	Caries experience associated with: At-risk of overweight lower family income Less educated parent Heavier mothers Higher soda pop intake by age Final prediction model: mother’s education and ‘at risk of overweight’	**Caries Prevalence**: 31% **BMI**: Underweight 3% Normal 72% At risk overweight 19% Overweight 5%
Martinez-Sotolongo & Martinez-Brito (2010)	Cuba	CS	649	8-13	The primary schools and one seconday school in Santa Marta, Varadero	3 unclear whether initial caries included	51	DFT/dft	Higher dental caries associated with higher BMI	**Caries Prevalence:** Normal weight: 41.77% Obese: 89.7% **BMI:** Obese: 37.3% Normal weight; 62.71% Underweight: 0%
Modeer et al. (2010)	Sweden	CC	130	10.3-18.3	Case-matched control study	1	10	Decayed surfaces DS(>0) DMFT indices	BMI-sds associated with Decayed surface (DS>0) OR 1.31 (unadjusted): Age, gender, chronic disease, medication, salivary flow, bleeding on probing visible plaque index, tooth-brushing infrequency (evening and morning), parental country of birth, and educational level No association between BMI-sds and DFT/DMFT	**Caries prevalence** not provided Obese: Mean dmfs:2.2 ( SD = 2.8) Mean dft:2.2 ( SD = 2.5) Mean ds:0.7 ( SD = 1.6) Control: Mean dmfs: 2.6 ( SD = 3.8) Mean dft: 2.1 (SD = 2.7) Mean ds:0.1 (SD = .4) **Mean BMI**: Obese: 36.8 (SD=5.8); Control: 19.7 (SD=2.4)
Reifsnider et al. (2004)	USA (Mexican-American sample)	L	104	1-2	Obese babies enrolled in Special Supplemental Nutrition Program for Women, Infants and Children	4	4	Ordinal: Caries free = 0, white spots =1, filling = 2, frank caries = 3	Higher dental caries associated with higher BMI Dental insurance, transportation issues, lack of knowledge of where to obtain dental care for children and mother's perception of the condition of her children's teeth	**BMI**: 20.3 (SD = not provided)
Sharma & Hedge (2009)	India	CS	500 sample of convenience	8-12	Department of Pedodontics and Preventive Children Dentistry, A.B Shetty Memorial Institute of Dental Sciences, Mangalore	2 – whether initial caries was included is not specified	134	DMFS/dmfs	Higher rates of dental caries (DMFS) in overweight and obese children than normal weight children. Underweight children had significantly higher DMFS rates (but not dfs) than normal weight, overweight and obese children. Overweight children had higher preference for fatty and sweet foods than normal weight children	**Mean DFMS (SD not provided):** Underweight: 3.11 Normal weight: 1.58 Overweight: 2.48 Obese: 2.85 **Mean dfs**: Underweight: 2.00 Normal weight: 2.14 Overweight: 4.79 Obese: 3.25 **BMI**: Underweight 8.6% Normal weight 58.4% Overweight or at risk for overweight 22.2% Obese 10.8%
Vazquez-Nava et al. (2010)	Mexico	CS	1160	4-5	Cohort study of children in three cities, Tampico, Madero, and Altamira in Mexico	3* with white spots coded as initial caries	57	deft, defs	Overweight and at-risk overweight children had higher caries prevalence than children who were not overweight Caries also associated with sugar consumption, bottle feeding, smoking at home and tooth brushing ≤ once per day	**Caries Prevalence** 17.9% 19.6% boys 16.4% girls **Mn deft:** Total sample = 1.08 (2.33) Normal weight= 0.70 (1.94) At -risk overweight =1.50 (2.57) Overweight = 1.51 (2.71) **Mn defs:** Total sample = 1.43 (3.28) Normal weight = 0.93 (2.64) At-risk overweight = 1.95 (3.49) Overweight= 2.04 (3.97) **BMI:** Normal weight: 53.7% At-risk overweight: 14.2% 17.1% girls 11.3% boys Overweight: 32.1%
Willershausen et al. (2007a)	Germany	CS	1290	6-11	5 elementary schools in a medium sized city	2	9	DF-T df-t	Higher rates of dental caries associated with higher BMI in both primary and permanent dentition Higher rates of caries also associated with Age (older), Gender (M), daily consumption of sweets	**Caries Prevalence**: 61.4% **Mn df-t** Underweight= 1.43 (2.02) Normal weight= 1.82 (2.41) Overweight= 2.3 (2.75) Obese= 2.21 (2.8) **Mn DF-T** Underweight= .38(1.28) Normal weight= 0.53 (1.2) Overweight= 0 .85(1.4) Obese= 0.82 (1.3) **BMI:** Underweight 3.6% Normal weight 74.7% Overweight, 11.8% Obese 9.7%
Willershausen et al. (2007b)	Germany	CS	2071	6-10	5 elementary schools in Mainz	2	9	DF-T df-t Dichotomised DF-t/df-t>0	Higher rates of dental caries associated with higher BMI in both permanent and deciduous dentitions; Age	**Caries Prevalence** 54.1% **Mn df-t + DF-T** ranged from 1.4– 2.6 **Mn df-t +DF-T** Underweight= 1.67 Normal weight = 2.15 Overweight = 2.64 Obese= 2.7 **BMI:** Underweight 6.8% Normal 76.4% Overweight 10.5% Obese 6.3%
Willershausen et al. (2004)	Germany	CS	842	6-11	4 elementary schools of diverse social background from single medium sized city	2	9	DF-T df-t	Higher rates of dental caries associated with higher BMI in both permanent and deciduous Gender (slightly higher cavities prevalence in boys, particularly in the DF-T-index and if overweight.	**Caries prevalence**: 63% **Mn DF-T** Normal weight=0.57 Overweight = 0.91 Obese = 0.88 **Mn df-t** Normal weight = 2.08 Overweight = 2.48 Obese = 2.23 **BMI:** Underweight 2.1% Normal weight 71.7% Overweight 12.0% Obese 13.3%

**Table 3:** Negative association between caries and BMI (higher caries associated with lower BMI)

**Authors**	**Country**	**Design**	**N**	**Age**	**Sampling**	**Dental rating**	HDI*	**Dental measure**	**Significant cariogenic risk factors**	**Sample demographics**
Benzian et al. (2011)	Philippines	CS	1951	11-13	Stratified cluster sampling (68 public schools)	3* at dentinal/cavity level	112	DMFT+dmft index PUFA+pufa (odontogenic infections) index Also categorised: dmft+DMFT >0 PUFA/+pufa <1; >1	Higher DMFT+dmft rates in underweight than normal weight. Higher PUFA+pufa index in underweight than normal weight. Final regression model found PUFA+pufa >0, Gender (M), No. siblings (>4) more likely to have low BMI PUFA/pufa index better predictor or BMI than DMFT/dmft Children with caries involving pulp or with odontogenic infection had increased risk of low BMI	**Caries Prevalence**: 82.3% Mn DMFT+dmft = 3.12 **Odontogenic infections**: 55.7% Mn PUFA+pufa= 1.15 **BMI:** Above normal: 1% Below normal: 27.1%
Cameron et al. (2006)	Scotland	CS	165 children with severe dental decay	3-11	Restricted: Children attending for extraction under GA	2	28	dmft dentine caries	Higher dmft in underweight children Higher dental caries also associated with Carstairs index (measure of social deprivation)	**Caries**: Mn dmft 7.9 (*SD=*3.5) **BMI**: stats not provided 71% children socially deprived
Floyd (2009)	Taiwan	CS	577	6	Two schools (affluent and less affluent) in Taipei	3	24	def	Higher caries (def) associated with lower BMI in less affluent group but not in affluent group. Housing and parental occupation for the less affluent sample were marginally associated (*p=*0.061 and 0.071 respectively)	**Mn def:** Less affluent group = 6.81(SD=3.66) Affluent group = 4.78(SD= 3.12) **BMI** : Less affluent group = 14.04 (SD=1.33) Affluent Group = 14.80 (SD=1.83)
Kopycka-Kedzierawski et al. (2008) a	USA	CS	10180	2-18	NHANES III (1988–1994) Nationally representative sample	2	4	DMFS and dfs dichotomised as either having caries experience or not Dfs and DFS in children aged 2–11 years were estimated	Age 6–11 years: Higher caries risk in Normal-weight children than: •at-risk or overweight (decid) •overweight (Perm) Age 12–18 years: Higher caries risk in Normal-weight children than: •overweight (Perm) Multiple logistic regression: 6–11 years of age, poverty, low level of education of household head and serum cotinine levels significantly associated with increased risk of caries experience in the primary dentition. Low level of education of household head, blood lead level above the median and other race/ ethnicity associated with increased risk of having caries experience in permanent dentition 12–18 years of age: Low level of education of household head and time since last dental visit also associated with increased risk of caries experience in permanent dentition	**Caries prevalence (%)**: 6-11yrs old (Primary dentition) Overall:49.5 (1.6) Overweight= 40.6 (4.7) At risk = 45.5 (4.6) Normal weight= 51.4 (1.6) Underweight = not reported 6-11yrs old (Permanent dentition) Overall:25.9 (1.7) Overweight= 17.6 (2.9) At risk = 29.9 (4) Normal weight= 26.5 (1.7) Underweight = not reported 12-18yrs (Permanent dentition) Overall: 66.3 (1.9) Overweight= 57.7 (4.6) At risk =67.8 (4.7) Normal weight= 67.2 (2.2) Underweight = not reported
Narksawat et al. (2009)	Thailand	CS	862	12-14	Quasi stratified sampling of 77 districts	3*	103	Prevalence DMFT =/>0	Thai Ministry of Public Health manual used to classify children as underweight, normal, overweight and obese. Inverse association between nutritional status and DMFT	**Caries prevalence**: 62.1% **Mn DMFT**: Overall sample = 1.93 (2.16) Normal weight: 2.03 (2.2) Underweight : 2.19 (2.19) Overweight: 1.23 (1.86) Obese: 0.89 (1.36) **BMI:** Obese: 6.3% Overweight: 5.3 % Normal :78.3% Underweight: 10.1%
Ngoenwiwatkul & Leela-Adisorn (2009)	Thailand	CS	212	6-7	Two primary schools	3 – cavity level	103	Dmfs index Prevalence dmfs =/>0	Higher DMFT index with decreased BMI Gender (Boys) for dental caries status (not dmfs) on primary dentition	**Caries Prevalence**: 80.2% **Mn dmfs** =12.4 (12.3) 70% of children experienced toothache **Mn BMI:** 15.5 (4.6) 45.8% participants in low percentile (5th<BMI-for- age<15th)
Olivira et al.(2008)	Brazil	CS	1018	1-5	Randomly selected from all children attending for vaccinations in 17 Health centres in city of Diadema	4	84	dmsf index Dichotomous dmfs =/>6	Mothers’ and fathers’ education level, household overcrowding, and number of children associated with dental caries prevalence. **For ECC (LR):** Caries prevalence and mothers’ education ,age of child, low BMI and clinically detectable dental plaque .The final hierarchical model demonstrated that mothers’ level of education, age, dental plaque were associated with and dental caries prevalence **For S-ECC** (LR): BMI not associated with severity Mothers’ education ,family income ,age lower weight-for-age and clinically detectable dental plaque related to dental caries severity	**Caries Prevalence**: 23.4% S-ECC(dmfs≥6) = 8.2%
Sanchez-Perez et al. (2010)	Mexico	L	110 with 88 at 4-year follow up	7-11	Public elementary school in middle-income area of Mexico City	3 – cavity level	57	dmft/DMFT dmfs/DMFS	Higher dmfs scores associated with lower SE level	**Caries Prevalence**: At age 7= 59.1% At age 8= 55.7% At age 9= 59.1% At age 10 = 50% At age 11= 40.9% **BMI**: Thin 25% Normal 45.5% At risk of overweight 12.5% Overweight 17%
Sharma & Hedge (2009)	India	CS	500 sample of convenience	8-12	Department of Pedodontics ad Preventive Children Dentistry, A.B Shetty Memorial Institute of Dental Sciences, Mangalore	2 – whether initial caries was included is not specified	134	DMFS/dmfs	Higher rates of dental caries (DMFS) in overweight and obese children than normal weight children. Underweight children had significantly higher DMFS rates (but not dfs) than normal weight, overweight and obese children.	**Mn DFMS**: Underweight: 3.11 Normal weight: 1.58 Overweight: 2.48 Obese: 2.85 **Mn dfs:** Underweight: 2.00 Normal weight: 2.14 Overweight: 4.79 Obese: 3.25 **BMI:** Underweight: 8.6% Normal weight: 58.4% Overweight/at risk: 22.2% Obese: 10.8%

**Table 4:** No Association between dental caries and BMI

**Authors**	**Country**	**Design**	**N**	**Age**	**Sampling**	**Dental rating**	HDI*	**Dental measure**	**Significant cariogenic risk factors**	**Sample demographics**
Cereceda et al. (2010)	Chile	CS	1190 ‘lower middle class’ sample	5-15	Stratified random sampling by gender and grade of eight primary schools from different districts of Santiago	3 at cavity level	44	COPD dmft	No association between caries and BMI	**Caries prevalence:** 79.5%. Caries prevalence by BMI: Underweight: 60% Normal weight: 80% Overweight: 78.1% Obese: 79.9% **BMI:** Underweight: 1.2% Normal weight: 51.6% Overweight: 25% Obese: 22%
Cinar et al. (2011)	Denmark	CS	332	15	Eight Danish municipalities selected for the purpose of representing various geographical areas of the Denmark and various degrees of urbanisation	2- cavity level (for 76% of the sample) 3- cavity level (for the rest of the sample)	16	DMFT	No direct association High loading on “health cluster” for BMI, DMFT, daily fruit consumption, and non smoking	**Caries prevalence = 62%** Mn DMFT: 2.03 (*SD=* 3.01) **BMI:** Mn BMI: 21.30 (*SD=*3.62) Obese 16%
Cinar & Murtomaa (2011)	Turkey	CS	611 360 public school 254 private school	10-12	Two schools selected by cluster sampling from high- and low- socio-economic level suburbs	3 – cavity level	92	DMFS	Attendance at public school associated with higher caries rates and lower rates of BMI DMFS, CPI and BMI shared the “health” cluster among both private and public school children	**Caries prevalence:** Public School: 91% Mn DMFS:4.44 (*SD=* 3.4) Private School: 70% Mn DMFS: 2.64 (*SD=* 2.6) **BMI:** Public School Obese: 25%; Non-obese: 75% Private School: 70% Obese: 40%; Non-obese: 60%
Cinar & Murtomaa (2008)	Finland and Turkey	CS	949 Finnish 338 Turkish 611	10 -12	Matched suburbs. Participating schools in Turkey selected through cluster sampling to represent socio-economic range of district.	1 Fin 3Turk – cavity level	22 92	DMFT	No direct association found between BMI and DMFT Turkish children from public school had lower mean BMI but higher Mn DMFT than Turkish children in private school Turkish sample higher in BMI and dental caries than Finnish sample. FA found obesity and caries shared same cluster.	**Caries Prevalence:** Finland: 33% Turkey: 84% **Mn DMFT:** Finland: 0.71 (*SD*= 1.54) Turkey 2.93 (*SD=* 1.99) **BMI** Finland: 20% obese Turkey: 28% obese Turkish private vs public schools: **Caries prevalence**: Public schools: 92%; Private: 73% **Obesity**: Public schools: 39% Private: 22%
de Carvalho Sales-Peres et al. (2010)	Brazil	CS	207	12	From eight schools (public and private) in the Midwest region of São Paulo	3 – cavity level	84	DMFT index	No association between caries and BMI Higher dental caries was associated with lower socioeconomic status	**Caries prevalence**: Private school: 11.9% Public school: 60.8% **Mn Dmft**: Private schools: 0.23 Public schools: 2.16 **BMI**: Private schools: Low weight: 35.59% Normal weight: 55.93% Overweight: 8.47% Obese: 0% **Public schools**: Low weight: 41.22% Normal weight: 52,03% Overweight: 4.73% Obese: 2.03%
D’mello et al. (2011)	New Zealand	CS	200 sample of convenience	3-8	High caries of high anxiety patient in the paediatric dentistry clinics at the University of Otago School of Dentistry	2	5	Dmft (number of deciduous decayed, missing and restored teeth)	No association between BMI and caries	**Caries:** Mn Dmft = 6.1 ( *SD* =3.7) Obese: 24% had dmft ≥8; Overweight: 37.5% had dmft≥8 35.4% had dmft ≥8 **BMI:** Obese: 17% (8.5%) Overweight: 23% (11.5%) Mn BMI = 16.0% (2.0)
Dye et al. (2004)	USA	CS	4236	2-5	NHANES III (1988-1994)	2	4	Dfs Dichotomous: dfs=/>0 Continuous 0, 1-2, 3-5, >6 surfaces untreated	No association between BMI and caries Higher Caries associated with: Low parental education achievement, Ethnicity (greater caries experience in Mexican-Americans than non-Hispanics) Poverty status (=/< 200% of the federal poverty level) Not receiving breastmilk Not eating breakfast daily Eating < 5 servings fruit & veg Not having dental visit within 12 months, Age	**Caries Prevalence** : 2 yrs: 7.7% 3 yrs: 15.5% 4 yrs: 29.6% 5 yrs: 40.2% **BMI:** Obese: 23% Overweight: 26.4% Normal & Underweight: 23.5%
Frisbee et al. (2010)	USA	CS	128	3-18	5 counties in West Virginia	5	4	Parent report – Now or ever had a cavity, filling, tooth pulled and overall dental health	No association between BMI and caries	**Caries prevalence:** Now or ever had a cavity: 61% Now or even had a filling,: 56% Now or even had a tooth pulled: 36% **Overall all dental health**: Excellent to very good: 55%; Poor to good: 45% **BMI:** Overweight or obese 56%
Granville-Garcia et al. (2008)	Brazil	CS	2651	1-5	84 private and public elementary schools in Recife (city)	3- cavity level	84	dmft	No association between caries and BMI Significantly higher decayed in public school.	**Caries Prevalence** 19% Public school: 26.4% Private school: 11.4% **dmft**: ~ 1.12 **BMI:** Obese: Overall 9% Public school: 4.6% Private school: 13.6%
Jamelli et al. (2010)	Brazil	CS with nested case control	689	12	Public school in the municipality of Caruaru; low socio-economic 465 cases (DMFT >0); *182 controls (DMFT=0) *no details on matching criteria	No details	84	DMFT	No association between caries and BMICaries associated with having visited a clinician.	**Caries Prevalence**: 71.8% Mn DMFT = 2.9 **BMI:** Low weight: 5.5% Risk of overweight: 9.3% Overweigh/Obesity: 3.2%
Juarez-Lopez & Villa-Ramos (2010)	Mexico	CS	189	3-6	Convenience sample from Iztapalapa´s area of Mexico City.	Information not provided	57	dmf-t; dmf-s	No association between dental caries and weight category (normal, overweight and obese). Gender (female)	**Caries prevalence**: Normal weight: 77% Overweight: 84% Obese: 79% **BMI:** Normal weight:33% Overweight: 33% Obese: 33%
Jürgensen & Petersen (2009)	Laos	CS	621	12	Multistage random sampling to select 10 representative elementary schools	3 cavity level	138	Cavity level dmft/DMFT	No association between dental caries and BMI Caries associated with semi-urbanisation, poor self-assessment of general health, often experiencing tooth ache in last 12 months, and several time being absent from school in last 12 months, higher economic status, gender (girls), impairment of quality of life (i.e., problems with eating, smiling and sleeping), dental visits in the last 12 months, acute dental visits, preference for intake of sweet drinks during school hours and low attitude level towards health	**Caries Prevalence**: 56% DMFT = 1.8 (SE=.09) dmft = .4 (SE=.04) **BMI:** Normal weight: 60% Overweight: 8% Underweight: 32%
Kopycka-Kedzierawski et al. (2008) a	USA	CS	10180	2-18	NHANES III (1988-1994) Nationally representative sample	2	4	DMFS and dfs dichotomised as either having caries experience or not Dfs and DFS in children aged 2-11 years were estimated	Age 2-5 years: No association between dental caries and BMI Caries risk associated with: poverty and Mexican–American Ethnicity, cotinine levels	**Caries prevalence (%)**: 2-5yrs old; Primary Caries Overall:23.8 (1.4) Overweight= 23 (3.6) At risk = 26.4 (3) Normal weight= 23.5-(1.4) 6-11yrs old; Primary Caries Overall: 49.5 (1.6) Overweight= 40.6 (4.7) At risk = 45.5 (4.6) Normal weight= 51.4 (1.6) 6-11yrs old; Permanent Caries Overall: 25.9 (1.7) Overweight= 17.6 (2.9) At risk = 29.9 (4.0) Normal weight= 26.5 (1.7) 12-18yrs; Permanent Caries Overall: 66.3 (1.0) Overweight= 57.7 (4.6) At risk =67.8 (4.7) Normal weight= 67.2 (2.2)
b	USA	CS	7568	2-18	NHANES 1999-2002 Nationally representative sample	2	4	DMFS and dfs dichotomised as either having caries experience or not Dfs and DFS in children aged 2-11 years were estimated	No association between dental caries and BMI at any age group Age 2-5 years: Caries risk associated with: Mexican–American ethnicity, poverty, time since the last dental visit and blood lead levels above median associated with increased risk 6–11 years of age: Caries risk associated with: Mexican–American ethnicity, time since the last dental visit, poverty and serum cotinine levels 12–18 years of age: Caries risk associated with: Mexican– American ethnicity, time since the last dental visit, poverty and serum cotinine levels	**Caries prevalence (%):** 2-5yrs old: Primary Caries Overall: 28.2 (1.8) Overweight= 35.7 (5.8) At risk = 24.3 (5.4) Normal weight= 27.7 (1.8) 6-11yrs old; Primary Caries Overall: 49 (2.5) Overweight= 52.3 (3) At risk = 42.5 (4.1) Normal weight= 49.7 (2.9) 6-11yrs old; Permanent Caries Overall: 20.3 (1.4) Overweight= 23 (2.4) At risk = 23.1 (3.2) Normal weight= 19.1 (1.7) 12-18yrs; Permanent Caries Overall: 56.8 (1.1) Overweight= 56.6 (2.7) At risk =58.2 (2.9) Normal weight= 56.6 (1.2)
Macek & Mitola (2006)	USA	CS	7617	2-17	NHANES 1999-2002 Nationally representative sample	2	4	Prevalence DMFT /dmft>0 Severity geometric mean for DMFT and dmft	No association between dental caries prevalence and weight categories Dental caries severity (geometric Mn DMFT) in permanent dentition associated with BMI: overweight children had lower geometric mean DMFT	**Caries prevalence (%):** 2-5 yrs (primary dentition) Overall: 27.5 (1.7) Underweight: 18 (5.2) Overweight: 36.1 (6.4) At risk of overweight: 26.9 (5.0) Normal: 28.1 (.8) 6-17 yrs (permanentdentition) Overall: 37.8(1.2) Underweight: 31.7(6.3) Overweight: 38.8(1.7) At risk of overweight: 38.1 (2.3) Normal: 37.8(1.4) **BMI:** Underweight: 4% At risk o/weight: 15% Overweight: 15% Normal weight: 63%
Moreira et al. (2006)	Brazil	CS	3330 (1665 obese; 1665 normal-weight)	12-15	Random sampling from public and private schools in plba	3 cavity level	84	DMFT	No association between dental caries and BMI Higher rates of dental caries associated in Public versus Private school, age ( 12 vs 15 yrs old)	**Caries Prevalence in:** Obese children: 30% Private Schools: 9.0% Public Schools: 50.9% Normal weight: 31% Private Schools: 9.6% Public Schools: 52.4% **Mn DMFT in** : Obese children in Private schools: 1.90 Public schools: 4.27 Normal-weight children in Private schools: 1.91 Public schools: 4.25
Pinto et al. (2007)	USA	CS	135 sample of convenience: 81% African American	M*=* 8.7( *SD* = 2.37)	Initial visit at (urban) Pennsylvania Dental School	2	4	DS/ds	No association between ds/Ds and BMI or between ds/Ds and gender or ethnicity Age significantly associated with Ds/ds	**Mn Ds score** 2.06 (CI 1.4-2.7) **BMI**: Mn BMI 18.36 (3.5) At risk overweight: 12% Overweight: 15%
Sadeghi et al. (2011)	Iran	CS	747	12-15	Twelve state and private secondary schools	3- cavity level	88	DMFT	No association between DMFT and BMI Males had higher DMFT than females	**Caries prevalence: 83.9%** **Mn DMFT**=2.83 (2.2) Underweight: 2.91 (2.2) Normal weight: 2.92 (2.3) At risk for overweight: 2.54 (1.8) Overweight: 2.34 (1.9) **BMI:** Underweight: 7.5% Normal weight: 72.8% At risk for overweight: 13.8% Overweight: 5.9%
Scheutz et al. (2007)	Tanzania	L 1997, 1999 and 2003	218	~6-14	Two primary schools (‘Affluent’ and ‘Poor’) in Dar es Salaam	3 cavity level	152	DMFS	No association between DMFS and low BMI	**Caries:** **DMFS** at baseline: Cohort 1= 0.33; Cohort 2= 0.37; Cohort 3= 0.32
Sheller et al. (2009)	USA	Retrospective case study	293 children with severe early childhood caries	2-5	Thirty different state, low income population	1	4	dmft Number teeth with Pulp involvement	No association between dmft and BMI Other factors associated with higher dmft/pulp involvement were Age (older) and ethnicity (Asian and ‘not reported’)	**Caries Prevalence:** 100% **Mn dmft**: =11.8 Underweight: 11.6 (1.5) Normal weight: 11.9 (.5) At risk for overweight: 11.1 (1.4) Overweight: 12.2 (1.4) **Mn pulp involved teeth** = 4.1 Underweight: 4.5 (1.5) Normal weight: 4.0 (.5) At risk for overweight: 4.0 (1.5) Overweight: 3.9 (1.0) **BMI:** Underweight: 12% Normal weight: 69% At risk for overweight: 9% Overweight: 11%
Tramini et al. (2009)^1^	France	CS	835	12	Randomly selected from Montpellier schools	3 caries in to dentine	20	DMFT	No association between DMFT and BMI Dental caries associated with higher sugar consumption, soft drink consumption and gender	**Caries prevalence:** 51.7% Underweight= 40% Normal weight = 51.7% Overweight= 50.5% Obese= 62.5% **Mn DMFT:** 1.47 Underweight= 0.73 Normal weight = 1.47 Overweight= 1.58 Obese= 1.66 **Mn BMI** = 18.9
Tripathi et al. (2010)	India	CS	2688	6-17	Selected from a private and two governments schools in Bareilly	3 cavity level	134	DMFT	No association between DMFT caries and weight category.	**Caries prevalence** = 19.1% Private schools= 27.6% Government schools = 9.6% **BMI:** Obese: 4.7% Private schools: Obese 7.5%; Non-obese 92.6% Government schools: Obese 1.57%; Non-obese 98.4%
Van Gemert-Schriks et al. (2011)	Suriname	CS	380	6	Seventeen schools from 2 different regions of the Rainforst, selected from the databases of the Medical Mission	3	104	Total caries experience (dmfs) Total-ds Dichotomised dentogenic infections >0/=0	No association between dmfs and BMI Higher rates of caries associated with reduced height suggesting caries is impacting on normal growth and development.	**Caries:** M total-ds: 14.0 (+/- 10.1)

^1^Authors found a negative association using a logistic and Poisson regression models but report no association after undertaking a zero-inflated and zero-inflated negative binomial regression models.

HDI* = Human Development Index.

**Table 5:** Comparison of sample demographics and method of dental examination for studies finding different associations between body mass index and dental caries

	**Positive association**	**Negative association**	**No association**
Sample	17	9	24
Median Human Development Index	10 (Mean = 21.9;	84 (Mean = 72.11;	50.50 (Mean = 55.33;
	*SD* = 32.9)	*SD* = 45.55)	*SD* = 49.69)
% Rank 1 to 2 dental examination	70.6	33.3	45.8
Median caries prevalence %	48.05 (Mean = 51.24;	59.1 (Mean = 58.03;	50.35 (Mean = 49.47;
	*SD* = 23.39)	*SD* = 21.59)	*SD* = 27.21)
Median dmft/DMFT	2.12 (Mean = 2.13;	3.11 (Mean = 4.69;	2.04 (Mean = 3.04;
	*SD* = 1.21)	*SD* = 3.41)	*SD* = 3.40)
Median % sample overweight	21.65 (Mean = 26.15;	20.55 (Mean = 18.77;	20 (Mean = 24.65;
	*SD* = 14.87)	*SD* = 15.10)	*SD* = 17.55)
Median % sample underweight	4.2 (Mean = 12.90;	25 (Mean = 23.32;	12 (Mean = 18.75;
	*SD* = 24.56)	*SD* = 15.11)	*SD* = 14.48)

Given that a number of studies used samples of convenience and special populations, (for example, hospital lists for dental extractions under general anesthetic [22] and lists for obesity programs [23]), the distribution of dental and BMI scores varies between studies. The results of these studies are of interest, but it is important to be mindful that these studies will have not tested the association between dental caries and BMI across the full range of potential scores. For this reason, sample statistics for caries and BMI measures (where provided in the studies) are included in summary tables for consideration when comparing results. The summary tables also provide information about each study’s setting, design, sample, dental measures, human development index (HDI), and factors associated significantly with dental caries in childhood and adolescence to facilitate interpretation of findings across studies.

After assessing for bias, only five studies met the criteria for A rating [19,24-26], and 20 studies met the criteria for a B rating (see Additional file 1). The findings of the five A-rated studies were mixed: two found that dental caries was higher in children with higher BMI [24,25]; one found a negative association in children aged 6 to 11 and 12 to 18 years but no association in children aged 2 to 5 years [19]; and two found no association in children aged 2 to 17 years [19,26]. Four of the five studies used data collected as part of the National Health and Nutrition Examination Survey (NHANES) database, a large nationally representative sample in the United States [19,25,26], and three studies used the same 1999 to 2002 cohort [19,25,26]. Hong *et al*. [25], Macek and Mitola [26], and Kopycka-Kedzierawski *et al*. [19] analyzed the data collected from children aged 2 to 5 years in the 1999 to 2002 cohort and reported different results: positive, no association, and no association, respectively. The sample sizes between these three studies differed slightly (1,506; 1,449; 1,719; respectively); Hong *et al*. [25] excluded children with fewer than 10 teeth, which might explain the smaller sample, but no exclusion criteria are provided for the other studies. All studies found similar trends for children at risk/overweight to have higher risk of dental caries than normal-weight children. Hong *et al*. [25] stratified by age and found the difference to be significant in children aged 60 to 72 months whereas Macek and Mitola [26] and Kopycka-Kedzierawski *et al*. [19] collapsed across age and found no association. Kopycka-Kedzierawski *et al*. [19] investigated other age groups within the 1999 to 2002 cohort and found no association in children aged 6 to 18 years.

The fourth study that used the NHANES III cohort [19] found that children aged 6 to 18 years who were overweight had a *reduced* risk of dental caries in both permanent and deciduous teeth compared to children of normal weight. No association was found in children aged 2 to 5 years. The fifth study [24] was a large cohort study from Sweden. A prospective association between obesity at an early age (4, 5, 7, and 10 years) and dental caries at age 12 years was reported. Thus, across these five A-ranked studies, four found trends consistent with a positive association between dental caries and BMI, which were significant in only two studies. The fifth found a negative association. One important problem with all five A-ranked studies is the restricted BMI range in the samples; underweight children were significantly under-represented. Only one study reported the proportion of underweight children in their sample (4.2%; [25]), and no studies included underweight as a BMI category. This is problematic for two reasons. First, the association between BMI and dental caries was inadequately tested because the association was not tested across the full range of BMI scores, and second, it must be concluded that any underweight children were absorbed within the ‘normal-weight’ category for comparison with children at-risk and overweight because no information was provided about their exclusion. If underweight children have systematically higher rates of caries than normal-weight children, and there is evidence that this may be the case (for example, [9]), caries incidence and severity in the normal-weight group would be inflated by the inclusion of underweight children, and potential differences between normal-weight and overweight-groups attenuated. The non-significant trends found in the NHANES studies [19,25,26] might be explained in this manner. Underweight children should be excluded from the analysis if insufficient in number to instantiate a comparison group, and it would be helpful if this information was provided.

#### Dental caries associated with higher BMI

Table 2 contains a summary of the 17 studies that found that dental caries prevalence or severity is higher in children and adolescents with higher BMI/body fat index. Caries prevalence ranged from 17.9% [27] to 90% [23] but the population-based studies reported prevalence rates between 42% [25] and 67% [5]. The dmft/DMFT rates were positively skewed, ranging from 1.4 [28] to 6.9 [23] with most studies reporting dmft/DMFT of approximately 2.0. The BMI distributions in this group appeared to be negatively skewed with seven studies [11,16,21,25,28-30] reporting that approximately 2 to 10% of their samples were underweight and 20 to 30% of their samples were overweight or obese. Six studies [23,24,27,31-33] appeared to have no underweight children in their samples, and remaining studies failed to provide sufficient detail [14,34,35]. The samples in these studies therefore appeared to be positively skewed for dental caries and negatively skewed for BMI.

##### Other factors associated with caries

Overall, the findings from this group of studies suggest that dental caries, as measured by prevalence or severity in deciduous and permanent teeth, increases with increased BMI. There is some suggestion that this association is moderated by age; three studies [25,28,31] found the association between high BMI and high dental caries appeared at around the age of 5 to 9 years and not earlier, although two [14, 25,] found the association in children under age 5 years. Other factors found to influence caries prevalence and severity rates include consuming ‘caries risk products’ more than three times per day at age 1 year [5]; sweets more than once per week at age 3 years [5]; higher rates of soda pop consumption [29,34]; sugar consumption [27]; lower socioeconomic status/poverty index/living in a disadvantaged neighborhood [24,25,29,34]; ethnicity [5], specifically being Hispanic or non-Hispanic black [25]; dental fatalism in caregivers [34]; parents’ poor attitudes to dental health and psychosocial factors [5]; less educated parent [29]; and heavier mothers [29].

#### Dental caries associated with lower BMI

Table 3 summarizes the nine studies that found a negative association between dental caries and BMI. Caries prevalence ranged from 23.4% [36] to approximately 80% [37], [38]. The dmft/DMFT rates ranged from 2.07 [16] to 14.0 [36]. Two studies reported odontogenic infections in more than half of the samples [37,38]. Three of the nine studies in Table 3 described the distribution of both underweight and overweight children in their sample [9,17,21].

##### Other factors associated with caries

Other factors associated with dental caries were social deprivation/affluence/lower socioeconomic level/lower household education level [9,19,39,40], all of which have also been associated with obesity [41], and larger families [36,37].

#### No association between dental caries and BMI

Table 4 summarises the 23 studies that found no association between dental caries and BMI. Caries prevalence ranged from 19.1% [19] to 91% [42] with higher prevalence in public schools (60.8% [43], 26.4% [44], 52.4% [45]) compared to private schools (11.9% [43], 11.4% [44], 9.6% [45]). Mean dmft/DMFT varied from .23 in private school children in Brazil [43] to 14 in children from remote areas of the rain forest of Suriname [46], with most studies reporting approximately 2.0. Seven of the 22 studies provided a breakdown of BMI categories, and the distributions included both a negative [26,47-49] and a positive skew [43,50,51]. Unfortunately, many studies described only the proportion of obese and non-obese participants. Failure to distinguish between normal-weight and underweight children makes it difficult to know how well the association between caries and BMI was tested in underweight children or between normal-weight and overweight chidlren. Proportions of obese children reported in these studies ranged from 20 to 50% [8,15,20,25,44,48,52].

##### Other factors associated with caries

Factors that were found to be associated with caries were ethnicity [8,47], low parental education achievement [8,19], poverty/lower socioeconomic level (below the federal poverty level [8,19,43]), higher SES level [50], female gender [48,53] not eating breakfast daily [8], eating fewer than five servings of fruit and vegetables daily [8], attending public school [20,42,44,50], school absenteeism [50], soft drink consumption [50,54], low health attitude [50], and reduced height [46].

##### Emergent patterns

Table 5 summarizes emergent patterns that may help account for the dispte findings between the three sets of studies. These patterns primarily relate to differences in the method of dental examination and sample demographics. These differences are noted below.

1. Method of dental examination: Studies that found a positive association between BMI and dental caries primarily used dental examinations that permitted the detection of initial caries (that is, ranked 1 or 2) whereas studies finding a negative, or no association, tended to use field examination methods (that is, ranked 3 or below), which underestimated caries rates (see Tables 2, 3 and 4 for detail and Table 5 for summary). A chi-square test of independence revealed this association approached significance (*χ*^2^ (2) = 5.19; *P* = .07).

2. Sample differences: Studies that found a positive association between BMI and dental caries were primarily conducted in Europe and the United States (see Table 2); those that found an inverse association were primarily conducted in Asia and South America (see Table 3); and those that found no association were largely conducted in the United States, South America and Europe. In an effort to quantify and compare the level of development of the countries represented in the three sets of studies, the Human Development Index (HDI) ranking for each sample is provided in Tables 2, 3 and 4. The HDI is a composite human development index that combines life expectancy, educational attainment and income to rank and compare the level of development of different countries [53]. A Kruskal-Wallis test revealed a significant difference in HDI between the three sets of studies (*χ*^2^ (2) = 7.067, *P* <.05) Those studies finding a positive association between dental caries and BMI used samples from more highly developed countries than studies that found an inverse association (*U* = 27.00, *P* <.01).

3. Prevalence: Dental caries prevalence tended to be similar across the three sets of studies although it should be recognized that studies summarized in Tables 3 and 4 tended to exclude non-cavitated lesions and therefore underestimate caries prevalence. Caries severity, as measured by dmft/DMFT tended to be higher in studies finding an inverse association between BMI and dental caries than studies finding a positive or no association. A Kruskal-Wallis analysis found a significant difference (*χ*^2^ (2) = 7.255, *P* <.05). Post hoc tests, using Mann Whitney U, suggests studies that found an inverse association between dental caries and BMI had involved samples with a significantly higher dmft/DMFT rate than studies finding no association (*U* = 67, *P =* 0.46) or a positive association (*U =* 73.50, *P =* 0.03).

4. BMI distribution: The nature of the distribution of BMI differed across the three sets of studies, which is problematic. In order to determine whether BMI is adequately tested against dental caries it is important to ensure that the sample represents the full range of BMI categories (that is, low (underweight), normal, and high (overweight/obese)). Only 68% of the studies reported the proportion of their sample that was overweight or obese, and 48% reported the proportion that was underweight. A Kruskal-Wallis test found underweight participants were significantly under-represented in studies that found a positive association between BMI and dental caries (*χ*^2^ (2) = 6.877, *P* <.05) compared with studies finding an inverse associations (*U*=8, *P* <.05), and no association (*U*=23, *P* = .046). No differences were found between studies in the proportion of the sample that was overweight or obese although only half of studies that found negative association reported the proportion of the sample that was overweight or obese.

A related issue is the inappropriate collapsing of groups across BMI categories as mentioned earlier. Studies that collapse across BMI categories assume a linear relationship exists between dental caries and BMI. If the relationship is non-linear, collapsing across BMI groups can attenuate any between-group differences between overweight or underweight children and the normal-weight reference group. Similarly, analyses that assume a linear relationship between BMI and dental caries, such as bivariate correlations may not detect a non-linear relationship. Given the evidence that dental caries is associated with both high and low BMI, analyses appropriate for non-linear associations might be more appropriate. Only four of the 23 studies that found no association between dental caries and BMI compared dental caries across low-weight, normal-weight, and over-weight groups [26,48-50]. Of the remaining 20 studies, five studies used analyses that assumed a linear relationship [43,46,47,52,55], and 11 studies compared overweight or obese children with ‘non-obese’ or ‘normal-weight’ children [8,15,19,22,43-45,51,54,56] without providing information about how they accounted for underweight children in their sample. Three studies excluded underweight children to permit a comparison between obese and normal-weight children [20,42,57].

### Discussion

The results of this systematic review show that there is still significant disagreement as to the existence and nature of an association between dental caries and BMI. Forty-eight percent of studies reviewed found no association between dental caries and BMI; 35% found a positive association and 19% found an inverse association. Our results are therefore consistent with those of Kantovitz *et al*. [10]; however, we would like to speculate further about the association between dental caries and BMI, offer possible reasons for the disparity in findings, and make recommendations for modifications to future research in this area. We suggest that dental caries and BMI are related in a nonlinear fashion with more dental caries occurring in individuals with either higher or lower BMI. Furthermore we suggest that methodological factors including sample demographics, the sensitivity of the dental examination, and the nature of the data analyses undertaken influence whether or not the association is detected.

The evidence supporting an inverse relationship between dental caries and BMI comes from studies in developing countries and/or from samples with severe dental caries. Severe dental caries may well reduce eating ability thereby resulting in poor weight gain. Evidence to support this can be seen when, post-comprehensive dental rehabilitation, young children with early childhood caries show significantly increased growth velocities compared with controls [58]. Malnutrition could also predispose to dental caries; deficiencies in protein or energy foods may lead to protein-energy malnutrition, decreased salivary flow, calculus formation, high levels of caries and reduced growth [42]. Chronic malnutrition, particularly during the early years, has been shown to increase susceptibility to dental caries in the primary dentition (for example, [59]) perhaps via enamel hypoplasia and salivary hypofunction [60]. Alternatively both outcomes could be influenced by a third variable (or cluster of variables) such as those associated with SES.

Socioeconomic factors clearly impact the development of caries and need to be understood. Lower socioeconomic status (SES), whether measured by living in a disadvantaged neighborhood, below the poverty line, attending public versus private school (for example [24,25,29]), is associated with higher caries indices. A cluster of factors such as low parental education level, ethnicity, limited access to services and support, associated with low SES, are also associated with higher caries rates (for example, [25,29,34]). However dental caries is not limited to those from low SES backgrounds; children from high SES groups, whose parents have high expendable income, can have increased exposure to fermentable carbohydrates and may be at an increased risk of dental caries (for example, [50,61]). Obesity is also experienced in children from higher SES backgrounds [2,7].

The studies that support a positive association between BMI and dental caries include those in which 1) samples were negatively skewed for BMI with underweight children underrepresented; 2) samples were from highly developed countries with high standards of living and improved access to public health (presumably including fluoride); and 3) studies that tended to use more sensitive dental examinations that permitted the detection of initial caries. It is possible that the development of dental caries in more affluent populations follows a different pathway to that in less-affluent populations. Dental caries is likely to be slower progressing (for example due to increased fluoride exposure or reduced likelihood of chronic malnutrition) and it may therefore be critical to include initial caries in caries diagnosis in more affluent populations in order to detect subtle differences in dental health.

It is not surprising to find that children who are overweight or obese also have relatively high levels of dental caries given that overweight children tend to consume high levels of soda [62] and other energy-dense foods [63], many of which are cariogenic and obesogenic. Modeer *et al*. [32] suggest that obese children are at risk of dental caries because they have reduced salivary flow, something also found in underweight children [60], and which is associated with protein-deficient malnutrition. Obese children may well suffer from protein deficient malnutrition if their energy intake is made up of high carbohydrate, highly processed foods.

Almost half of the studies included in this review found no association between dental caries and BMI. We have suggested that several methodological factors may have contributed to these findings including: 1) a failure to include initial caries in caries assessment, which is important given the possibility that the ratio of initial to manifest caries may be different in overweight and underweight samples; 2) a failure to appropriately sample from the full range of BMI scores to adequately represent underweight, normal, and overweight and obese participants; 3) the undertaking of analyses that assume a linear relationship; and 4) the collapsing across BMI categories when making between-group comparisons (for example [64]). Costacurta *et al*. [16] suggest that BMI may not be the best measure of body fat composition when testing the association between dental caries and obesity. They suggest that misclassification of childhood obesity using BMI might account for the failure of studies to detect the association between child adiposity and dental caries (in samples where children are normal-weight and overweight).

Based on our findings in this review we can offer several recommendations for future research in this field:

1. When screening for dental caries, initial caries should be included in the caries measure, and the same diagnostic criteria should be used by all researchers to enable comparisons to be made across studies. Despite attempts to standardize caries assessment for research purposes since 2002 (for example, ICDAS, 2009 [65]) considerable disparity in diagnostic criteria still exists.

2. To permit some level of comparison to be made across studies, it would be useful to have access to details about sample pmeters for target variables such as BMI and caries pmeters, prevalence rates, a breakdown of BMI groups and demographic information such as SES.

3. The possibility of a non-linear association between dental caries and BMI should be considered, and appropriate analyses performed when testing the relationship.

4. Prospective longitudinal studies are needed to explore the causal relationships between the variables alluded to in this systematic review and to inform interventions. Longitudinal studies are also required to study the long-term association between dental health indices and broader general health outcomes because dental indices may provide a more reliable indicator of future health outcomes than BMI. BMI fluctuates throughout life and may not, at any given point in time, provide an accurate representation of a lifetime of dietary and health behaviors. Also, it is important to establish first, the time required for the association between obesity and caries to manifest, and second, to determine whether the association changes over time.

5. Given the impact of parent factors such as socioeconomic status, education level, diet, dental fatalism, health attitudes and so on, on the development of dental caries as found across studies, a focus on familial or parental influences is warranted. Such an approach might help explicate the effect of wider ecological influences, such as SES on the development of dental caries, especially in early childhood when dietary habits are being formed and implemented by parents.

### Conclusions

There is evidence that dental caries is associated with both high and low BMI. Although the precise nature of these associations remains unclear, it is possible that different factors are involved in the development of caries in children with high and low BMI and in high and low socio-economic strata. Evidence supports the proposal of combined strategies to target both dental caries and obesity simultaneously, however further investigation of the association between the diseases and among their predictors is required. Specific attention should be given to longitudinal studies to gauge the association between early childhood caries and health outcomes in adolescence and adulthood, to the inclusion of younger children (aged 0 to 6 years) in the samples, to the perseverance of dietary and health-related behaviors developed during the preschool years, and to parental or familial influences on the development of these patterns. Abbreviations: BMI, body mass index; DMFT/dmft, decayed (D/d ), missing (M/m), filled (F/f) surfaces (S/s) or teeth (T/t) index in the permanent/primary dentition; HDI, human development index; NHANES, National Health and Nutrition Examination Survey; SES, socioeconomic status.

### Competing interests

The authors have no competing interests to declare.

### Authors’ contributions

CB carried out the initial database searches and preliminary readings. MH re-read abstracts and articles for final selection and prepared the draft. All authors read and approved the final manuscript.

## Supplementary Material

Additional file 1 Review of included studies’ methodology. Click here for file

Additional file 2 Excluded papers with reasons. Click here for file
