# Supplementary material for: Body mass index and dental caries in children and adolescents: a systematic review of literature published 2004 to 2011
Source: Syst Rev. 2012 Nov 21;1:57. doi: 10.1186/2046-4053-1-57 (PMC3621095; doi:10.1186/2046-4053-1-57)
Supplement: Additional file 1 — Review of included studies’ methodology. [file 2046-4053-1-57-S1.docx]

|  |  | Review of included studies’ methodology | | | | | | | |
| --- | --- | --- | --- | --- | --- | --- | --- | --- | --- |
|  | Quality assessment | | | | Statistical analyses and results | | | Rating | |
|  | **Representativeness of sample** | Assessment/control of important and/or additional confounders | **whether BMI objectively reported** | **quality of caries assessment** | | **Was association between caries and BMI tested across low-, normal, -high BMI** | **Magnitude of association** | |  |
| Alm et al. (2011) | 2 | 2 | 1 | 1 | | No: Between-groups analysis : Underweight and Normal weight children combined | 6 yrs old group:  Risk of caries experience (Obese vs low-normal weight)  OR=2.5; 95% CI: 1.0-5.9 | | B |
| Alm (2008) &  Alm et al. (2008) | 2 | 2 | 1 | 1 | | No: Between-groups analysis :  Underweight and Normal weight children combined | Not reported | | B |
| Bailleul-Forestier et al. (2007) | 4 | 2 | 1 | 3 | | No: Between-groups analysis :  Underweight and Normal weight children combined | Not reported | | D |
| Benzian et al. (2011) | 1 | 2 | 1 | 3^a^ | | No: Between groups: over-weight and normal weight combined  Regression; assumes linear relationship | Not reported | | B |
| Cameron et al. (2006) | 4 | 2 | 1 | 2 | | No: No information about BMI distribution | Not reported | | C |
| Cereceda et al. (2010) | 2 | 1 Age and gender | 1 | 3 | | Yes LogReg caries yes/no against low- normal and high BMI | Non sign. results | | B |
| Cinar et al. (2011) | 1 | 2 | 2  Parent report | 2 | | No: but underweight excluded to permit comparison between obese and non-obese | Non sign. Results | | B |
| Cinar & Murtomaa (2011) | 2 | 1 Type of schools (private vs public) and life-style factors (daily consumption of milk at breakfast, regular bedtime on school nights, and recommended tooth-brushing) | 2 | 3 | | No: but underweight excluded to permit comparison between obese and non-obese | Non sign. results | | C |
| Cinar & Murtomaa (2008) | 2 | 1 Lifestyle factors and leisure time (TV viewing on a school day; regularity of family dinner; fizzy & fast food per week; daily snacking on fruit b/t meals), and type of school (public and private school in the Turkish sample | 2 | 1 Fin  3Turk | | No: but underweight excluded to permit comparison between obese and non-obese | Non sign. results | | B Fin  C Turkey |
| Costacurta et al. (2011) | 4 | 1 Age and gender | 1 | 1 | | Yes Between-groups  No: correlation assumes linear association | r = 0.221 dmft;  r= 0.237 DMFT | | B |
| de Carvalho Sales-Peres et al. (2010) | 2 | 2 | 1 | 3 | | No: Model assumes linear association | Non sign. results | | C |
| D’Mello et al. (2011) | 4 | 1 Gender & ethnicity | 1 | 2 | | No: Underweight not reported | Non sign. results | | C |
| Dye et al. (2004) | 1 | 2 | 1 | 2 | | No: Underweight not reported | Non sign. results | | B |
| Floyd (2009) | 2 | 1 Parental occupational category (labor, business, or whitecollar) and housing (older single-story row housing vs. newer multi-story dwellings) to the less affluent sample regression model | 1 | 3 | | No: Regression model assumes a linear relationship |  | | B |
| Frisbee et al. (2010) | 4 | 2 | 1 | 5 | | No: Underweight not reported | Non sign. results | | D |
| Gerdin et al. (2008) | 1 | 1 Age, gender and socioeconomic status | 1 | 1 | | No: Underweight not reported | For the LMR:  Childhood BMI at 4 years of age: b-value 0.048; SE: 0.020  Childhood BMI at 5 years of age: b-value 0.050; SE:0.018  Childhood BMI at 7 years of age: b-value 0.032; SE:0.013  Childhood BMI at 10 years of age: b-value 0.024; SE: 0.009 | | A |
| Granville-Garcia et al. (2008) | 1 | 1Type of school (private vs public) | 1 | 3 | | No: Underweight not reported | Non sign. results | | B |
| Hilgers et al. (2006) | 4 | 1Age & gender | 1 | 1 | | Yes | Not reported | | B |
| Hong et al. (2008) | 1 | 1Age and race | 1 | 2 | | Yes Between-groups  No: Multivariate predictive models assumed linear association | Not reported | | A |
| Ismail et al. (2009) | 3 | 1 | 1 | 2 | | No: Model assumes linear association | Not reported | | B |
| Jamelli et al. (2010) | 2 | 2 | 1 | No details | | No: Model assumes linear association | Non sign. results | | 5+ |
| Juarez-Lopez & Villa-Ramos (2010) | 4 | 2 | 1 | No details | | No: Underweight not reported | Non sign. results | | 7+ |
| Jürgensen & Petersen (2009) | 1 | 2 | 1 | 3 | | Yes Between-groups | Non sign. results | | B |
| Kopycka-Kedzierawski et al. (2008) | 1 | 1  NHANES III, the logistic regression adjusted for sex, race/ethnicity, geographic  region of the country, poverty status, level of  education of the household head, time since last  dental visit, and blood lead and cotinine levels  NHANES 1999–2002: logistic regression analyses adjusted for sex, race/ethnicity, last dental visit, poverty status,  blood lead levels (below and above median) and  serum cotinine levels. | 1 | 2 | | No: Underweight not reported | **Cohort III**  Ref group: Normal weight  6-11yrs primary caries OR (MLR):  Overweight: 0.7 *p =*0.04  At risk:0.7  *p =*0.04  6-11yrs permanent caries OR (MLR):  Overweight: 0.6 *p =*0.03  At risk: Non sign. results  12-18 yrs OR (MLR):  Overweight: 0.5 *p =*0.02  At risk: Non sign. results  **Cohort 1999-2002**  Not sign at all ages | | A  A |
| Macek & Mitola (2006) | 1 | 1 Age, sex, race/ethnicity, poverty status | 1 | 2 | | Yes Between-group analysis  Multiple logistic regression | Non sign. results | | A |
| Marshall et al. (2007) | 1 | 1 Age, child’s cumulative fluoride intake, mother’ education | 1 | 3 | | No: Underweight not reported | Predicting caries experience:  Ref group: No caries  Children ‘at risk’ of overweight OR 3.02 (CI 1.46, 6.25) | | B |
| Martinez-Sotolongo & Martinez-Brito (2010) | 2 | 1Age and gender | 1 | 3 | | No: Underweight not reported | Obese vs Normal weight against caries risk: OR = 28.2 (CI 15.7- 88.2) | | B |
| Modeer et al. (2009) | 4 | 1 Parental country of birth, and educational level | 1 | 1 | | No: Underweight not reported | Caries experience DS (>0) as  dependent variable: BMI-sds (P = 0.002, OR = 1.31)  MLR:  OR for BMI after analysis is adjusted for  range of variables ranged OR 1.24-1.36 | | B |
| Moreira et al. (2006) | 1 | 2 | 1 | 3 | | No: Underweight not reported | Non sign. results | | B |
| Narksawat et al. (2009) | 1 | 1 Brushing frequency, use of fluoride toothpaste, having more than 3 snacks a day, having more than 2 dental visits a year, receiving oral health education every 3 months, residential area (urban/rural), age, gender | 1 | 3^a^ | | Yes Between-groups  Yes Multiple Logistic regression | OR for MLR:  Risk caries experience of underweight vs overweight/obese (OR= 2.22; 95% CI= 1.20-4.09) Normal vs overweight/obese: (OR= 1.915; 95% CI=1.20-3.00) | | B |
| Ngoenwiwatkul & Leela-Adisorn (2009) | 4 | 1Gender and number of dental visits in MLR | 1 | 3 | | No: Underweight not reported | OR adjusted:  Risk of underweight: Caries vs no caries  OR = 1.031 (95% CI = 1.005-1.058) | | C |
| Oliveira et al. (2008) | 2 | 1Mother’s education level | 1 | 4 | | Yes Logistic regression | Risk caries Underweight: OR=3.20 1.31-7.78) Overweight: OR=.60 (0.36-1.00) | | C |
| Pinto et al. (2007) | 3 | 2 | 1 | 2 | | No: Underweight not reported | Not significant | | C |
| Reifsnider et al. (2004) | 4 | 1Mother's perception of condition of children's teeth, lack of transportation for dental care and Oral Health Needs Assessment scale (OHNA) | 1 | 4 | | No: HMR model assumed a linear relationship | Pearson b/t ECC & BMI; r=0.20  hierarchical multiple regression (adjusted R^ =.21; F=7.13;  p=.00). | | D |
| Sadeghi et al. (2011) | 2 | 2 | 1 | 3 | | Yes Between-groups  No: Model assumes linear association | Non sign. results | | C |
| Sanchez-Perez et al. (2010) | 4 | 1Age at baseline, gender, number of primary teeth, and SE stratum | 1 | 3 | | Yes Between-groups | dmfs at baseline compared to dmfs at follow up:  Risk of being overweight (baseline) –0.12 (follow up –2.30) *p =* 0.032  Overweight: (baseline) ––2.01 (follow up –2.69) *p =* 0.035 | | C |
| Scheutz et al. (2007) | 4 | 1 Lactobacilli, mutans streptococci, stimulated saliva flow rate, school, gender | 2 | 3 | | No: Overweight not reported | Non sign. results | | C |
| Sharma & Hegde (2009) | 4 | 2 | 1 | 2 | | Yes Between-group | Not reported | | C |
| Sheller et al. (2009) | 4 | 1 Age, gender, ethnicity, Medicaid status in the adjusted multiple regression model | 1 | 1 | | No: Model assumes linear association | Non sign. results | | B |
| Tramini et al. (2009) | 2 | 1 Gender, sugar consumption, soft drink consumption, type of school | 1 | 3 | | No: Underweight underrepresented (1.8%) | Logistic regression predicting caries experience: BMI:  1.05 (1.00–1.10)  Poisson model  1.03 (1.01–1.05) | | B |
| Tripathi et al. (2010) | 4 | 1Type of school (private vs public) | 1 | 3 | | No: Underweight and normal weight combined | Non sign. results | | C |
| Van Gemert-Schriks et al. (2011) | 4 | 2 | 1 | 3 | | No: Model assumes linear association | Non sign. results | | D |
| Vazquez-Nava et al. (2010) | 2 | 1  Adjusted for gender, sugar consumption, smoking in the home, bottle feeding, tooth brushing frequency  gender, sugary product | 1 | 3 ^a^ | | No: Underweight not represented | At-risk of caries at-risk of overweight (adjusted OR = 1.94; 95 percent CI = 1.30-2.89) and overweight children (adjusted OR = 1.95; 95 percent CI = 1.42-2.64) | | B |
| Willerhausen et al. (2007a) | 4 | 1 Age and gender | 1 | 2 | | Yes but underweight underrepresented (3.6%) | Not reported | | C |
| Willershausen et al. (2007b) | 4 | 2 | 1 | 2 | | Yes | Not reported | | C |
| Willershausen et al. (2004) | 4 | 1 Gender | 1 | 2 | | No: Underweight and normal weight combined | Not reported | | C |

Samples that involved forms of stratified or cluster sampling of countries or districts that ensured representation of a range of SES values were rated 1, samples that represented cities or towns using some form of cluster sampling (e.g., of schools) were ranked 2, samples of convenience with some randomisation involved in selection of participants were ranked 3, and sample of convenience without a random selection of participants were ranked 4.
