# Supplementary material for: Body mass index and dental caries in children and adolescents: a systematic review of literature published 2004 to 2011
Source: Syst Rev. 2012 Nov 21;1:57. doi: 10.1186/2046-4053-1-57 (PMC3621095; doi:10.1186/2046-4053-1-57)
Supplement: Additional file 2 — Excluded papers with reasons. [file 2046-4053-1-57-S2.docx]

Excluded papers with reasons

| **#** | **Authors** | **Reason** |
| --- | --- | --- |
| 1 | Al-Ansari et al. (2006) | Does not measure the association between caries and BMI |
| 2 | Arora et al. (2011) | No measure of BMI |
| 3 | Bastos et al. (2007) | No measure of BMI |
| 4 | Batista et al. (2009) | Sample age 5-53 |
| 5 | Bimstein & Katz (2009) | Review paper |
| 6 | British Dental Journal (2005) | Editorial |
| 7 | Clarke et al. (2006) | Does not measure the association between caries and BMI |
| 8 | Conrey et al. (2009) | Does not measure dental caries |
| 9 | Cronin et al. (2008) | Adult sample |
| 10 | Di Renzo et al. (2010) | Only abstract available |
| 11 | de Silva-Sanigorski et al. (2011) | No measure of BMI |
| 12 | Dostal & Jakusova (2007) | Does not measure the association of dental caries and BMI |
| 13 | Ekuni et al. 2008 | Participants aged between 18-24 (18>) |
| 14 | Enwonwu et al. (2004) | Adult sample |
| 15 | Fisher-Owens et al. (2007) | Does not measure the association between caries and BMI |
| 16 | Gravina et al. (2006) | Does not measure the association between caries and BMI |
| 17 | Godlewski et al. (2008) | Review paper |
| 18 | Gomber & Dewan (2006) | Does not measure the association between caries and BMI |
| 19 | Hisaw et al. (2009) | Editorial (guidelines for dentists) |
| 20 | Huang et al. (2009) | Does not measure the association between caries and BMI |
| 21 | Huang et al. (2006) | Does not measure the association between caries and BMI |
| 22 | Karjalainen (2007) | Does not measure the association between caries and BMI |
| 23 | Keels et al. (2008) | Does not measure the association between caries and BMI |
| 24 | Kelishadi et al. (2010) | No measure of BMI |
| 25 | Kumar et al. (2009) | Sample over 18 years |
| 26 | Lanigan et al. (2007) | Review paper |
| 27 | Liu et al. (2010) | Only abstract available |
| 28 | Locker (2005) | No measure of BMI |
| 29 | Malek et al. (2009) | Does not measure the association between caries and BMI |
| 30 | Mapengo et al., 2010 | Does not measure the association between caries and BMI |
| 31 | Marciani et al. (2004) | Does not measure the association between caries and BMI |
| 32 | Masson et al. (2010) | Does not measure the association between caries and BMI |
| 33 | Maupome et al. (2010) | Does not measure the association between caries and BMI |
| 34 | Mcguire et al. (2009) | Measure dental erosion, not dental caries |
| 35 | Mobley et al. (2009) | Review paper |
| 36 | Modeer et al. (2011) | Does not measure dental caries |
| 37 | Mota-Sanhua et al., 2008 | Does not measure the association between caries and BMI |
| 38 | Moynihan (2005) | Does not measure the association between caries and BMI |
| 39 | Moynihan (2005) | Editorial paper |
| 40 | Nasreddine et al. 2009 | Does not measure oral health/dental caries |
| 41 | Nicolau et al. (2005) | No measure of BMI |
| 42 | Nunn (2006) | Editorial paper |
| 43 | Ohlund et al., 2007 | No measure of BMI |
| 44 | Oziegbe et al. (2009) | Does not assess dental caries |
| 45 | Palmer et al. (2010) | Editorial paper |
| 46 | Palmer (2005) | Editorial paper |
| 47 | Peres et al. (2005) | No measure of BMI |
| 48 | Psoter et al. (2008) | Measures early childhood protein-energy malnutrition (EC-PEM) and not BMI |
| 49 | Reeves et al. 2006 | Participants aged between 13 to 21 years (18>) |
| 50 | Reid (2004) | Sample over 18 years |
| 51 | Sanders & Slade (2010) | No measure of BMI |
| 52 | Sanders et al. (2005) | Sample over 18 years |
| 53 | Schroth et al. (2009) | No measure of BMI |
| 54 | Sheiham (2006) | Review paper |
| 55 | Sohn, 2009 | Review paper |
| 56 | Tavares & Chomitz,(2009) | Does not measure the association between caries and BMI |
| 57 | Telford et al. (2011) | No measure of BMI |
| 58 | Touger-Decker & Mobley (2007) | Review paper |
| 59 | Triković-Janjić et al. (2008) | Does not measure the association between caries and BMI |
| 60 | Zermeño-Ibarra et al. (2010) | Does not assess dental caries |
| 61 | Vann et al. (2005) | Guidelines for Paediatricians |
| 62 | Zhou et al. (2011) | No measure of BMI |
